# Effects of probiotic (*Lactobacillus plantarum* and *Bacillus subtilis*) supplementation on mortality, growth performance, and carcass characteristics of native Vietnamese broilers challenged with *Salmonella* Typhimurium

**DOI:** 10.14202/vetworld.2022.2302-2308

**Published:** 2022-09-26

**Authors:** Tran Van Be Nam, Luu Huynh Anh, Huynh Tan Loc, Chau Thi Huyen Trang, Nguyen Thiet, Ly Thi Thu Lan, Tran Hoang Diep, Nguyen Hong Xuan, Nguyen Trong Ngu

**Affiliations:** 1 Department of Molecular Biotechnology, Biotechnology Research, and Development Institute, Can Tho University, Can Tho City, Vietnam; 2 Department of Animal Sciences, College of Agriculture, Can Tho University, Can Tho City, Vietnam; 3 Department of Veterinary Medicine, College of Agriculture, Can Tho University, Can Tho City, Vietnam; 4 Department of Agricultural Technology, College of Rural Development, Can Tho University, Can Tho City, Vietnam; 5 Animal Husbandry and Veterinary Medicine Department, School of Agriculture and Aquaculture, Tra Vinh University, Tra Vinh City, Vietnam; 6 Department of Animal Science and Veterinary, Faculty of Agriculture and Food Technology, Tien Giang University, My Tho City, Vietnam; 7 Food Technology Department, Faculty of Biological, Chemical, and Food Technology, Can Tho University of Technology, Can Tho City, Vietnam

**Keywords:** *Bacillus subtilis*, growth, *Lactobacillus plantarum*, mortality, Noi chicken

## Abstract

**Background and Aim:**

Probiotic species have been proven to be beneficial on broiler performance; however, most studies have focused on industrial chickens with fast growth, whereas little information concerning the use of these species on native chickens is available. This study aimed to investigate the effects of probiotics *Lactobacillus plantarum* (LP) and *Bacillus subtilis* (BS) on the mortality, growth rate, and carcass characteristics in native Noi chickens challenged with *Salmonella* Typhimurium.

**Materials and Methods:**

We divided 420 1-day-old Noi chicks into seven different treatment groups (n = 60): negative control (no *S*. Typhimurium, no probiotics or antibiotics); positive control (PC, *S*. Typhimurium infection, no probiotics or antibiotics); and *S*. Typhimurium infection and supplementation with LP, BS, LP + BS, enrofloxacin, and commercial probiotics, respectively. Treatment was for 96 days, and the chicks were orally challenged with *S*. Typhimurium at 22 days old.

**Results:**

No deaths occurred during the 4 weeks post-infection in the negative control, LP, or LP+BS groups. The PC group had the highest mortality rate (20%). Re-isolation of *S*. Typhimurium from the liver, spleen, and heart showed reduced bacterial counts at 1 week post-infection in the LP, BS, and LP + BS groups. The lowest body weight gain was observed in the PC group (949 g/bird), and chicks in the LP group gained 1148 g/bird. An improved feed conversion ratio was noted in the groups receiving probiotic supplementation (3.42–3.50 kg feed/kg gain). There was little evidence that probiotics affected carcass percentage and related parameters, such as breast, thigh and drumstick, and wings.

**Conclusion:**

*Lactobacillus plantarum* or BS dietary supplementation to native Noi broilers resulted in a lower mortality rate and improved body weight gain but did not affect carcass characteristics.

## Introduction

The global poultry industry plays an important role in providing protein-rich meals, such as eggs and meat, to consumers. However, its expansion has led to increased bacterial infections, which impact public health as well as cause substantial economic losses [1], of which salmonellosis is a problematic issue. Some predominant serotypes of *Salmonella* can cause serious clinical diseases, including gastroenteritis, enteric fever (typhoid fever), and bacteremia, in animals and humans [2, 3]. Over the past decades, antimicrobials have been extensively used in intensive poultry production to prevent and treat diseases as well as promote growth and productivity. Van Boeckel *et al*. [4] reported an expected increase of 67% in the use of antimicrobials in livestock and poultry farming, primarily due to the expanding worldwide demand for animal protein [5]. In Vietnam, antimicrobials were found in 5.4% of all chicken feed formulations, and a total of ten feed items for hens included one or more antimicrobials that might be used in the feed formulation [6]. The inappropriate use of antibiotics leads to negative issues, including microbiota disruption, antibiotic residues, and the development of antibiotic-resistant bacteria, which may infect poultry products and seriously threaten human health [7, 8].

Recently, the use of nonantibiotic additives in poultry feed has been expanded in an effort to enhance development and feed utilization because antibiotic use in animal feed was prohibited. Feed additives, including acidifiers, antioxidants, probiotics, and prebiotics, have been effectively utilized in poultry production due to a strong drive to find alternatives to antibiotics [2]. Thus, probiotics have become prominent supplements and food additives and are generally used to promote healthy digestion since they have been demonstrated to improve growth performance [9] and prevent and manage enteric infections in poultry [10].

Bacteria from the genus *Bacillus* are widely used in animal production to improve weight gain and feed efficiency and can be easily administered to animals as an oral dose. The *Bacillus* genus comprises gram-positive facultative anaerobes. Their ability to form endospores enables them to survive longer at elevated temperatures or pressures than non-spore-forming bacteria. Probiotic bacteria, such as *Bacillus subtilis* (BS), improve animal performance and health conditions by rebalancing the intestinal microbiota [11]. *Bacillus* strains isolated and characterized from poultry feces have been reported to reduce *Salmonella* Typhimurium from the crop and ceca of broiler chickens in experimental infections [12]. The *Bacillus* genus is common in birds and can improve health by reducing harmful microorganisms [13]. In addition, *Lactobacillus* is an excellent probiotic for domestic animals because it inhibits the growth of pathogenic bacteria while promoting the development of nonpathogenic microorganisms via the production of various metabolites, thereby improving the gut microbiota [14]. Wu *et al*. [15] concluded that *Lactobacillus plantarum* (LP) promoted broiler intestinal and body health condition by improving intestinal mucosal barrier function, antioxidative ability, and immunity while decreasing cell apoptosis with strain-specificity.

Most of the above-mentioned studies mainly focused on fast-growing broiler chickens, and information on the use of probiotics in native broilers with a long-life span remains limited. Therefore, we aimed to investigate the preventive efficacy of BS and LP against *S*. Typhimurium in Noi broilers, an indigenous Vietnamese chicken breed, with regard to mortality, *S*. Typhimurium reduction, growth performance, and carcass characteristics. Treatments with single or combined strains of probiotics were also evaluated to determine their efficiency for long-term use in native broilers in comparison with antibiotic use.

## Materials and Methods

### Ethical approval

The chickens were cared for and handled according to the Animal Husbandry Law (32/2018/QH14) of Vietnam, and the experimental protocol was approved by the Council of College of Agriculture, Can Tho University (A10-01-2021/KNN).

### Study period and location

The study was conducted from March to July 2021 on the Can Tho University’s experimental farm in Phung Hiep District, Hau Giang province.

### Salmonella, antibiotics, and probiotics

We obtained the virulent strain *Salmonella enterica* subspp. *enterica* serovar Typhimurium from the American Type Culture Collection (ATCC 14028; Manassas, Virginia, USA) for the experimental infection of Noi chickens. The antibiotic (enrofloxacin) and commercial probiotics (BS, 10^9^–10^10^ Colony-forming unit (CFU)/g; *Lactobacillus* spp., 10^6^–10^9^ CFU/g; and *Saccharomyces cerevisiae*, 10^6^–10^9^ CFU/g) were supplied by a local veterinary medicine company. Two probiotic strains, LP and BS, were isolated from fermented food and intestines of chicken raised at different poultry farms in the Mekong Delta of Vietnam and screened for probiotic characteristics [16].

### Birds and housing

All birds were vaccinated as instructed by the chicken breeding company. During the finishing stage, they were placed in confinement houses at a density of 10 birds/m^2^. A commercial broiler diet was provided *ad libitum*. The starter diet contained 16% crude protein and metabolizable energy of 2800 Kcal/kg, while the grower and finisher diet contained 14% crude protein and metabolizable energy of 2800 Kcal/kg. Drinking water was freely available.

### Experimental design

We used 420 1-day-old Noi broiler chicks of mixed sex to determine the preventive effect of probiotics against *S*. Typhimurium challenge. The chicks were randomly selected from a breeder flock and divided into seven different treatment groups (n = 60 birds each, four replicates per treatment): negative control (NC, no *S*. Typhimurium, no probiotic or antibiotic supplementation); positive control (PC; *S*. Typhimurium infection, no probiotic or antibiotic supplementation); and *S*. Typhimurium infection and supplementation with LP, BS, LP + BS, enrofloxacin (Enro, Can Tho, Vietnam)*,* and commercial probiotics (CProbi, Can Tho, Vietnam), respectively. The chickens were orally challenged with 5 × 10^7^ CFU/mL of *S*. Typhimurium [17] at 22 days old (average body weight, 176 g). The supplementation with corresponding probiotics (10 g/kg feed, 10^7^–10^8^ CFU/g) or antibiotics (10 mg/kg feed) started 2 days before infection and occurred twice a week until the end of the experiment.

### Data collection and measurements

We recorded the feed intake and body weight of each group weekly. Then, after adjusting for any dead birds, we calculated the feed conversion ratio (FCR) as feed intake (g)/weight increase (g). Mortality was monitored daily during the 4 weeks post-infection. We randomly selected four birds per treatment group (sex balanced when applicable) for *S*. Typhimurium re-isolation on days 7, 14, 21, and 28 post-infection and at the end of the experiment using the procedures of Gomes *et al*. [18]. At the end of the experiment, when chickens were 98 days old, we euthanized 56 broilers (8 per treatment, sex balanced) to assess carcass features and organ measurements. The broilers were individually weighed before euthanization through cervical dislocation and exsanguination. The breast, thigh and drumstick muscle, and wings were collected according to Goliomytis *et al*. [19]. We also removed and weighed the liver, spleen, heart, and gizzard, and the weights of the organs were calculated as a proportion of the body weight.

### Statistical analysis

The data were analyzed using the General Linear Model in Minitab 16.2.1 software (State College, Pennsylvania, USA). Tukey comparison test was used to determine the mean differences between treatments. p ≤ 0.05 was considered statistically significant.

## Results

**Figure-1:**
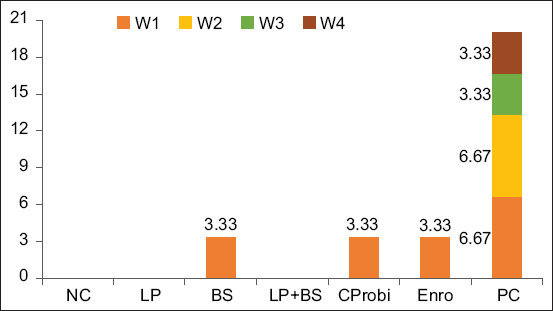
Mortality rate of chickens during 4-week post-infection with *Salmonella* Typhimurium. W=Week, NC=Negative control, LP=*Lactobacillus plantarum*, BS=*Bacillus subtilis*, Enro=Enrofloxacin, CProbi=Commercial probiotics, PC=Positive control.

Probiotic administration decreased the mortality rate of chickens challenged with *S*. Typhimurium (Figure-1). There were no deaths in the NC, LP, or LP + BS groups. The highest mortality rate (20%) was observed in the PC group, in which the majority of birds died within the first and second weeks post-infection. In addition, the results revealed that the efficacy of BS (3.33%) was similar to antibiotics and commercial probiotics provided in the diet.

**Figure-2:**
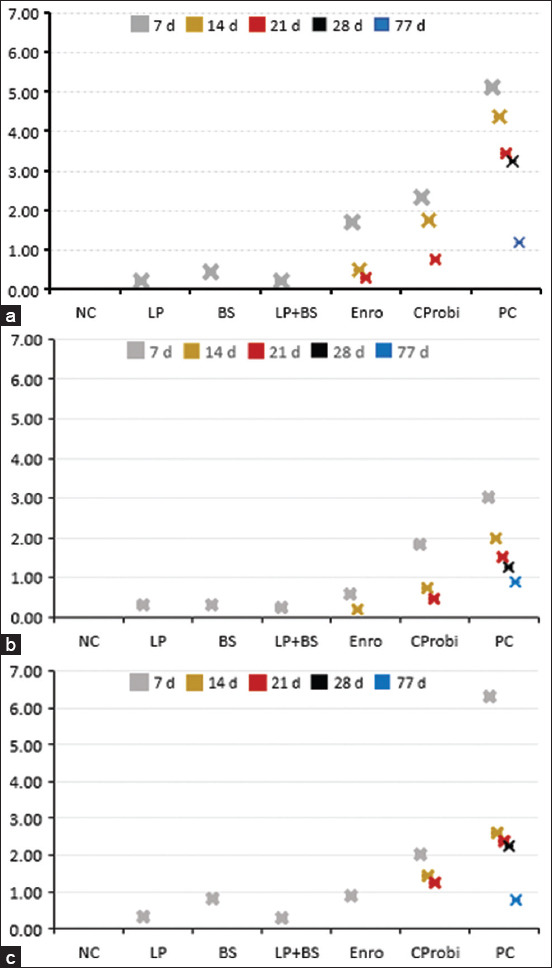
Bacterial counts in organs of chickens at different days’ post infection: (a) Liver, (b) spleen, (c) heart. NC=Negative control, LP=*Lactobacillus plantarum*, BS=*Bacillus subtilis*, Enro=Enrofloxacin, CProbi=Commercial probiotics, PC=Positive control.

Re-isolation of *S*. Typhimurium in several internal organs indicated a general tendency that, regardless of the number of days post-infection, the PC group had the highest bacteria count, followed by the Enro and CProbi groups (Figure-2). *S*. Typhimurium was found in all organs 7 days after infection, although there was a drop in density in birds treated with either probiotics or antibiotics. However, 1 week later, no *S*. Typhimurium was detected in the LP, BS, and LP + BS groups. Except for the PC group, all groups were free of *S*. Typhimurium in all examined organs at day 28 post-infection. In addition, in the PC group, the amount of *Salmonella* re-isolated from the heart was slightly higher than that re-isolated from the liver and spleen during the 4 weeks post-infection.

Table-1 shows the feed intake, body weight gain, and FCR of the different treatment groups. The addition of probiotics resulted in a significantly increased feed intake LP, 4,066 g/bird) between 22 and 98 days of age (p < 0.05), but the inclusion of enrofloxacin had no effect when compared with the positive control (3,774 g/bird). Improved body weight gains were also observed in treatments with LP and BS probiotic supplementation (1,098–1,155 g/bird), resulting in a reduced FCR.

**Table-1:** Effects of probiotic supplementation on growth performance of Noi broilers.

Parameters	Treatments							SEM	p-value

	NC	LP	BS	LP+BS	CProbi	Enro	PC		
Feed intake, g									
22–42 days	834	886	845	840	802	852	803	21.4	0.122
43–70 days	1,633^a^	1,522^ab^	1,403^b^	1,541^ab^	1,519^ab^	1,481^ab^	1,517^ab^	36.2	0.009
71–98 days	1,719^a^	1,658^ab^	1,613^abc^	1,598^abcd^	1,554^bcd^	1,435^d^	1,455^cd^	36.7	0.000
22–98 days	4,186^a^	4,066^ab^	3,861^ab^	3,979^ab^	3,874^ab^	3,768^b^	3,774^b^	74.7	0.003
Body weight gain, g									
22–42 days	293^ab^	284^b^	286^b^	322^a^	280^b^	285^b^	280^b^	6.62	0.001
43–70 days	485^a^	430^bc^	395^cd^	456^ab^	425^bc^	388^cd^	351^d^	11.5	0.000
71–98 days	420^a^	435^a^	417^a^	376^ab^	368^ab^	333^b^	318^b^	17.9	0.000
22–98 days	1,198^a^	1,148^abc^	1,098^bc^	1,155^ab^	1,072^cd^	1,005^de^	949^e^	18.0	0.000
FCR, kg feed/kg gain									
22–42 days	2.85^ab^	3.12^a^	2.97^ab^	2.61^b^	2.87^ab^	3.00^ab^	2.87^ab^	0.09	0.013
43–70 days	3.37^b^	3.55^b^	3.58^b^	3.38^b^	3.58^b^	3.84^ab^	4.33^a^	0.13	0.000
71–98 days	4.11	3.83	3.90	4.27	4.29	4.37	4.60	0.18	0.075
22–98 days	3.44^b^	3.50^b^	3.48^b^	3.42^b^	3.58^ab^	3.73^ab^	3.93^a^	0.08	0.002

NC=Negative control, LP=*Lactobacillus plantarum*, BS=*Bacillus subtilis*, Enro=Enrofloxacin, CProbi=Commercial probiotics, PC=Positive control, FCR=Feed conversion ratio, SEM=Standard error of the mean. ^a,b,c,d,e^ Within a row, values with different superscripts differ statistically at p < 0.05

The carcass characteristics and percentages of internal organs are presented in Table-2. Remarkably, the LP+BS group showed a significant improvement in carcass weights (960 g/bird) compared with that of the PC group (777 g/bird) (p < 0.05). There was also an increase in the ratio of liver and spleen to body weight in chickens receiving probiotic treatments. Moreover, even higher ratios were observed in the PC treatment, where chickens suffered from the *S*. Typhimurium infection but were not treated.

**Table-2:** Effect of probiotic on carcass characteristics and internal organs of chickens.

Parameters	Treatments							SEM	p-value

	NC	LP	BS	LP+BS	CProbi	Enro	PC		
BW, g	1,368^a^	1,336^ab^	1,296^bc^	1,325^ab^	1,242^c^	1,182^d^	1,121^e^	12.9	0.000
CW, g	971^a^	957^a^	923^b^	960^a^	888^c^	822^d^	777^e^	6.72	0.000
Carcass, % BW	71.0^ab^	71.6^ab^	71.3^ab^	72.4^a^	71.5^ab^	69.5^b^	69.4^b^	0.50	0.001
Breast, % CW	18.1^ab^	17.6^b^	17.3^b^	19.6^a^	18.3^ab^	18.4^ab^	18.3^ab^	0.44	0.023
Thigh + drumstick, % CW	22.6	21.6	22.5	23.2	23.2	22.6	23.1	0.63	0.607
Wing, % CW	13.5	14.0	13.0	13.8	14.6	13.5	14.2	0.42	0.215
Organ weight, % BW									
Liver	1.74^c^	2.33^ab^	1.92^bc^	2.11^abc^	2.02^abc^	2.13^abc^	2.40^a^	0.10	0.001
Spleen	0.18^b^	0.22^ab^	0.22^ab^	0.26^ab^	0.17^b^	0.16^b^	0.29^a^	0.02	0.002
Gizzard	2.01^ab^	1.84^b^	1.71^b^	1.82^b^	1.83^b^	2.02^ab^	2.48^a^	0.11	0.001
Heart	0.35	0.38	0.39	0.40	0.44	0.38	0.42	0.02	0.148

NC=Negative control, LP=*Lactobacillus plantarum*, BS=*Bacillus subtilis*, Enro=Enrofloxacin, CProbi=Commercial probiotics, PC=Positive control, SEM=Standard error of the mean, BW=Body weight, CW=Carcass weight. ^a,b,c,d,e^ Within a row, values with different superscripts differ statistically at p < 0.05

## Discussion

Alternatives to antibiotic use in the poultry industry are necessary to mitigate the spread of antimicrobial resistance. Since probiotics are beneficial bacteria with antibacterial and growth-promoting properties, we sought to identify and evaluate the effects of indigenous *Lactobacillus* and *Bacillus* species on native Noi broilers. In general, the inclusion of isolated LP and BS positively impacted most examined indicators. Our findings revealed that the PC group (*S*. Typhimurium-challenged, without probiotics or antibiotics) showed the highest mortality rate, which is consistent with earlier findings. El-Shall *et al*. [20] reported that broiler chickens challenged with *Salmonella* had the highest mortality rate (25%), and it was highest in the first- and second-weeks post-infection. The severity of clinical signs was worse in the birds that were *Salmonella*-challenged but received no probiotics than in those that received probiotics. Abd El-Ghany *et al*. [21] also reported the highest morbidity and mortality rates in the *Salmonella*-challenged group in their study. Moreover, in our study, enrofloxacin treatment did not result in improved prevention of *S*. Typhimurium infection, most likely because the bacteria were resistant to this antibiotic since enrofloxacin is one of the main treatments for *S*. Typhimurium administered to live chickens [22].

Sharma *et al*. [23] found *S*. Typhimurium in the spleen and liver at 16 weeks post-infection. In our study, *S*. Typhimurium was found in these organs, as well as the heart, at 11 weeks post-infection, but it was eliminated in the groups receiving probiotic treatments. A similar report by Dina and Hams [24] also confirmed a decline in the total bacterial count of *S*. Enteritidis isolated from challenged birds treated with probiotics. This could be because the production of lactic acid by the probiotic might have been unfavorable for *Salmonella* growth. A significant reduction in the incidence of *S*. Typhimurium has been observed 24 h after lactic acid bacteria administration, suggesting the potential of probiotics to control the gut microbiota and environmental factors, thereby limiting *Salmonella* colonization of birds [25]. Probiotics maintain the correct balance of beneficial microbial populations in the intestine of birds, and this is essential for efficient feed conversion, growth, productivity, and stimulation of immune mechanisms to combat pathogens [26]. In addition, probiotic activity in the gastrointestinal tract improves nutrient absorption, theoretically resulting in more energy being available for net energy production [27]. In our study, probiotic-supplemented treatments, either as a single or combination dose of LP and BS, resulted in improved feed intake, body weight gain, and FCR. Notably, the advantageous effects were even more pronounced when compared with antibiotic supplementation. We found that enrofloxacin supplementation was not very effective, as evidenced by the fact that the chickens in this group experienced lower weight gains and higher FCR that were almost identical to the results of those in the PC group. Supplementing broilers’ diet with a single probiotic or a mixture of probiotics significantly improves growth performance and decreases FCR under normal, stress, disease, and other conditions [14]. According to Abudabos *et al*. [2], BS affects growth parameters in broilers exposed to *S*. Typhimurium at the starter phase, where, in terms of growth performance and feed consumption, probiotic supplementation was as equally beneficial as antibiotic supplementation. In addition, some probiotic strains increased immunoglobulin A, G, and M levels in broilers, contributing to enhanced growth performance and disease resistance [27]. Collectively, this improved performance of chickens fed probiotics may be attributed to increased feed digestibility, maintaining a favorable gut microbiota, and promoting intestinal integrity. However, the impact of probiotic supplementation on broiler performance by differences in probiotic dose, bacteria species, feed type, and microbiomes requires further investigation.

We found little evidence that probiotics significantly affect carcass percentage and related parameters, such as breast, thigh and drumstick, and wings. There were no variations in carcass percentage between the control and probiotic-supplemented groups. In line with our findings, Qorbanpour *et al*. [28] observed that dietary treatments with multistrain probiotics (*L. acidophilus*, *L. casei*, *Enterococcus* *faecium*, and *Bifidobacterium thermophilum*) did not affect the weights of the carcass, breast, and thigh of chickens. However, Mehr *et al*. [29] reported that higher levels of probiotic supplementation resulted in heavier body and carcass weights and a higher percentage of breast weight.

In our study, most internal organ weight percentages were slightly higher in the PC group, except for the heart, where the weight proportion did not differ among the treatment groups. We also observed that the groups treated with probiotics showed an increasing trend in the percentage weight of the liver and spleen compared with the negative control. These outputs correlated fairly well with reports that the supplementation of broilers’ diets with BS significantly increased the relative weight of the spleen but not the liver [30, 31]. In other studies, the impact of probiotics on internal organ weight is unclear since no variations in the percentage weights of the liver, spleen, gizzard, and heart were observed between broilers fed control or a probiotic-supplemented diet [32, 33]. Overall, the above findings indicate that the effect of probiotics on the weight of visceral organs in chickens is unclear. To this end, future studies should focus on the type and quantity of microbial strains employed as probiotics and the metabolic activity of probiotics.

## Conclusion

Our study clarified that dietary supplementation with two strains of probiotics is likely to contribute to poultry health and increase growth performance in chickens. *Lactobacillus plantarum* or BS supplementation reduced the mortality rate and positively affected body weight gain and FCR, as well as decreased the count of *S*. Typhimurium in the liver, spleen, and heart of Noi broilers. On the other hand, there was minimal evidence that probiotics affected carcass percentage or related parameters. In addition, compared with antibiotic administration, the benefits of probiotics were significantly more apparent. Although the combination of the two probiotics did not exhibit a greater favorable influence on most of the parameters examined, they are a viable alternative to antibiotics in poultry production, particularly in native Noi broilers.

## Authors’ Contributions

TVBN, LHA, and NTN: Designed the experimental procedures. TVBN, LHA, HTL, NT, LTTL, and THD: Performed the experiments. TVBN, CTHT, NHX, and NTN: Interpreted the data and prepared the manuscript. All authors have read and approved the final manuscript.
